# Down and High: Reflections Regarding Depression and Cannabis

**DOI:** 10.3389/fpsyt.2021.625158

**Published:** 2021-05-14

**Authors:** Catherine Langlois, Stéphane Potvin, Atul Khullar, Smadar Valérie Tourjman

**Affiliations:** ^1^Faculty of Medicine, Université de Montréal, Montreal, QC, Canada; ^2^Department of Psychiatry and Addictology, Faculty of Medicine, Université de Montréal, Montréal, QC, Canada; ^3^Research Center of the Institut Universitaire en Santé Mentale de Montréal, Montréal, QC, Canada; ^4^Department of Psychiatry, University of Alberta, Edmonton, AB, Canada; ^5^Department of Psychiatry, Institut Universitaire en Santé Mentale de Montréal, Montréal, QC, Canada

**Keywords:** cannabis, depression, legalization, impact, epidemiology, mechanisms of action

## Abstract

In light of the recent changes in the legal status of cannabis in Canada, the understanding of the potential impact of the use of cannabis by individuals suffering from depression is increasingly considered as being important. It is fundamental that we look into the existing literature to examine the influence of cannabis on psychiatric conditions, including mood disorders. In this article, we will explore the relationship that exists between depression and cannabis. We will examine the impact of cannabis on the onset and course of depression, and its treatment. We have undertaken a wide-ranging review of the literature in order to address these questions. The evidence from longitudinal studies suggest that there is a bidirectional relationship between cannabis use and depression, such that cannabis use increases the risk for depression and vice-versa. This risk is possibly higher in heavy users having initiated their consumption in early adolescence. Clinical evidence also suggests that cannabis use is associated with a worse prognosis in individuals with major depressive disorder. The link with suicide remains controversial. Moreover, there is insufficient data to determine the impact of cannabis use on cognition in individuals with major depression disorder. Preliminary evidence suggesting that the endogenous cannabinoid system is involved in the pathophysiology of depression. This will need to be confirmed in future positron emission tomography studies. Randomized controlled trials are needed to investigate the potential efficacy of motivational interviewing and/or cognitive behavioral therapy for the treatment of cannabis use disorder in individuals with major depressive major disorder. Finally, although there is preclinical evidence suggesting that cannabidiol has antidepressant properties, randomized controlled trials will need to properly investigate this possibility in humans.

## Introduction

Depression is a leading cause of disability in the world (1, 2) with a lifetime prevalence in the general population of about 15% (3). As such, any factor that modifies the course or presentation of depression has a disproportionate impact on disability and individual burden of illness.

Cannabis is a widely used substance with pleotropic effects and has been proposed both as a treatment for and as a cause of depression. Cannabis is composed of 60–500 different compounds including a class of chemicals called cannabinoids (4–6); of these, delta-9-tetrahydrocannabinol (THC) and cannabidiol (CBD) are the most examined. THC is considered to be the main psychoactive component of cannabis (4), while CBD is purported to contribute to many of its therapeutic benefits (3).

The balance of harmful and therapeutic effects of CU in depression has not yet been clarified (7).

This review aims to synthesize the literature pertaining to the relationship between depression and cannabis use (CU). Particular attention to the potential mechanisms involved in this association will be considered.

## Prevalence of Cannabis Use

Cannabis is one of the most used substances worldwide (8). After alcohol and tobacco, cannabis ranks first for used substances in the United States (US) (9, 10) and Canada (11). Three to five percent of the world's population have used cannabis at least once (12, 13). Approximately 8 million Americans use cannabis every day or nearly every day (14).

CU is widespread among younger individuals with 7.6 million users in the 18–25 age group and 1.6 million in the 12–18 group in the US in 2017–2018 (15). A 2017 Canadian survey showed that the prevalence of past year cannabis use was higher in adolescents (19%) and in young adults under 25 years old (33%) than in adults over 25 (13%) (11). In the US, 60 percent of those who use cannabis for the first time are under 18 (16). In fact, in adolescents, the prevalence of CU has surpassed that of cigarette smoking (16).

The definitions of substance use disorders differ across systems of diagnostic classification (17). DSM-IV requires 3 or more of 7 criteria which include the presence of withdrawal, tolerance, use of larger amounts or over a longer time, repeated attempts to quit or control use, much time spent using, physical or psychological problems related to use and activities given up to use (17). DSM-5 requires only 2 of the 7 criteria for dependence or the DSM-IV criteria of abuse which include hazardous use, social or interpersonal problems related to use and neglected major roles in order to use (17). In DSM-IV, substance abuse included the criterion of legal problems as a consequence of use; this was eliminated in DSM-5 (17). The differing definitions relating to CU may contribute to variable results found in the literature.

The risk of developing dependence is about 9% and rises to 16% if CU is initiated in adolescence (18). Preliminary evidence suggests that the addiction potential of cannabis may depend on its THC content (19). The THC content of cannabis has increased from historic levels of 3–5% to the current levels of 25% (4, 5), potentially increasing the risk of addiction.

The prevalence of CU has increased with a prevalence in the US of 4.1% between 2001–2002 and 2012–2013 and 9.5% respectively (20). Over the same period, cannabis use disorder (CUD) prevalence in the US went up rose from 1.5 to 2.9% (21, 22).

## Context and Impact of Legalization

In the US, Colorado and Washington were the first states to make recreational use and sale of cannabis legal in the United States in 2014, although medical marijuana had already been legalized in Colorado since 2000 (23). Following legislation for recreational use, past-year CU in people 18 and older increased from 15% (2008–2009) to 24% (2015–2016) (24). Intriguingly, the impact of legalization on adolescent CU is less clear with some jurisdictions showing increased and others decreased use (12). Thus, factors other than legalization may also play a role in the change of prevalence of CU after its legalization. Canada legalized recreational cannabis in October 2018 (12, 25). In the year following legalization in Canada, increases of CU were noted with over a half million first time users, the most substantial increase being in men aged 45–64 years (25). One year on, a survey conducted by Statistics Canada documented an increase in past 3 month use from 14.9 to 16.8% (26) but decrements in adolescents aged 15–17. The same survey found that reported daily use increased only in those 65 and older.

The frequent use of cannabis is associated with a plethora of negative health and social consequences (14, 22). Where this issue has been studied, an increase in related consequences has occurred concurrently with an increase in CU in the states that have legalized medical cannabis (14). These negative consequences include increases in the prevalence of serious mental illness (14) and emergency department consultations for cannabis-related mood disorders, as well as suicide and intentional self-harm (12, 27, 28). It is important to underline that potentially positive effects of CU, such as decreased anxiety, have not been systematically studied (28). As legalization becomes more widespread, it becomes pressing to evaluate the consequences of the subsequent increased consumption in vulnerable populations such as those suffering from mood disorders (7).

## Potential Mechanisms Underlying the Relationship Between Cannabis and Depression

The endogenous cannabinoid (or endocannabinoids) system (ECS) (29) is involved in regulating functions such as mood, cognition, feeding behavior, pain perception, inflammation, and stress responses (8, 30). Furthermore, there is evidence that a hypoactive ECS may contribute to depression in humans (6).

The activity of the ECS is mediated by at least two cannabinoid receptors (CB1 and 2) and endogenous cannabinoids [2-arachidonoylglycerol (2AG) and anandamide] (31). THC is a partial agonist of CB1 and CB2, although its psychoactive effects derive from its activity on CB1 receptors (6). The CB1 receptor is widely expressed in regions which are involved in reward and cognitive functions (30). The CB1 receptor modulates the GABAergic, glutamatergic, serotoninergic and noradrenergic systems (5, 6, 8) and promotes myelination (32).

The ECS is further involved in the modulation of the hypothalamic pituitary axis (HPA) and brain derived neurotrophic factor (BDNF) (6, 33). The ECS also modulates inflammation: CB1 activation decreases inflammation through astrocytes, and CB2 through microglia (34). Importantly, these systems are also involved in the pathophysiology of depression (35–37).

There role of the ECS in the pathophysiology of depression is supported by several lines of evidence. For instance, CB1/CB2 receptor gene polymorphisms are associated with the behavioral characteristics typical of depression (38, 39). In rodents, CB1 receptor deficiency provides a model for depression and genetic modifications reducing its expression are associated with depressive behaviors and vulnerability to stress or social defeat (40). *In vivo* electrophysiological studies in rats have shown that acute or chronic low-dose stimulation by a full or partial agonist of the CB1 receptor produces an activation of the serotonin (5-HT) neurons in the dorsal raphe nucleus and increases their firing rate. On the other hand, sub-chronic or long-term high-dose stimulation by a CB1 receptor agonist causes an important decrease in the firing rates of the 5-HT cells of the dorsal raphe nucleus (41). Increases in firing rates of these neurons are seen with the administration of antidepressants and are considered to be an essential mechanism of action underpinning their therapeutic effects (42).

In animal models, the effect of low-doses of CB1 receptors agonists on the firing rate of 5-HT cells of the dorsal raphe nucleus is associated with antidepressant and anxiolytic effects, in contrast with high-doses which are associated with depressant effects (43). Lower doses of cannabinoids have antidepressant and anxiolytic effects while higher doses have the opposite effect (4). The effect of THC on dopamine release follows a similar biphasic pattern with low doses enhancing dopamine synthesis and high doses decreasing it (44). The dopaminergic system has been implicated in the pathophysiology of depression and in particular anhedonia (45) and it is possible to speculate that recreational use of low dose cannabis may generate mild euphoria while high dose cannabis may lead to anhedonia. In humans, the cerebrospinal fluid of individuals with depression is characterized by a reduction of endocannabinoid precursor levels (38).

CU leads to widespread alterations in cerebral function (46). In a meta-analysis examining the residual effects of CU on cognitive function following abstinence, functional imaging in cannabis users reveals decreased activations in the anterior cingulate cortex and dorsolateral prefrontal cortex (46). These changes were correlated with cognitive deficits (46). These same regions are involved in the pathophysiology of depression and are targeted by neuromodulation treatments of depression (47).

Partial agonism at the CB1 receptor is considered to mediate, at least in part, the behavioral and abuse potential of cannabis (48, 49). In humans, activation of CB1 receptors may lead to a reduction of l-DOPA induced dyskinesia (48), adding to the evidence that this receptor modulates the dopaminergic system. Clinical use of medications that target the CB1 receptor has led to the symptomatic relief of nausea, vomiting, loss of appetite and muscular spasticity, and there is interest in their potential anxiolytic and antidepressant effects (50). In humans, antagonism of the CB1 receptor can precipitate the onset of depression and suicidal ideation (38). Indeed, in trials using the CB1 receptor antagonist rimonabant to treat excess weight, symptoms of anxiety and depression were more frequent in the experimental than the placebo group (43). Rimonabant and a similar agent, Taranabant (43), were removed from the market due to the emergence of depression and suicidal ideation (38).

The cumulative weight of the evidence is that the ECS and cannabinoids play a role in the pathophysiology of depression and have a potential role in its treatment.

## Relationship Between Cannabis and Depression: Prevalence Data

The prevalence of depressive disorders is high in cannabis users (25%). Risk factors include female gender and earlier age of onset of use (51). The prevalence of major depressive disorder (MDD) in those with cannabis dependence (CD), CUD, and cannabis abuse (CA) is ~6.9, 4.7, and 1.0%, respectively (52). This highlights the importance of exploring the relationship between recreational, medical and heavy cannabis use (including CUD) and depression (53). Further, a meta-analysis published in 2021 found that the odds ratio (OR) for MDD comorbidity varied with the type of CU. The odds ratio was 4.83 for MDD-CD comorbidity, 2.60 for MDD-CUD comorbidity and 2.37 for MDD-CA comorbidity (52). An older 2014 meta-analysis of longitudinal studies found an OR of 1.17 for developing depression in cannabis users compared to controls. The same meta-analysis calculated an OR of 1.62 of developing depression in heavy cannabis users compared to non-users/light users (54). Another meta-analysis of longitudinal and case-control studies found that compared to non-regular use, regular use was associated with 1.5-fold odds of developing a MDE (29). Finally, a third meta-analysis found a unidirectional risk (OR = 1.33) of developing depression in adolescent and young cannabis users (55). In contrast, a study by Turna et al. found no difference between low (<1 g/day) and moderate users (1–2 g/day) (56). This observation may indicate a non-linear relationship between the degree of cannabis exposure and the risk of developing MDD; thus, low or possibly moderate use confers little risk of developing MDD, while heavy use is likely to lead to the emergence of depression. A recent systematic review of the impact of cannabis on the onset of mood disorders concluded that CU was associated with an increased risk of later depression (57). The same correlation was also observed in studies which focused on adolescents (57). Further, in a meta-analysis of longitudinal studies published in 2019, CU in adolescence was associated with a higher likelihood of developing depression in young adulthood (OR = 1.37) (58). Some studies show that the impact of CU may be greater in women who seem to be at higher risk of subsequently developing depression (59, 60). Early and frequent CU was associated with MDD in a large twin study (61). The duration of CUD is also associated with the emergence of comorbid mood disorders, including MDD (62), adding to the evidence that the degree of exposure to cannabis is related to depression. The frequent absence of linkage between infrequent or low dose CU and the emergence of depression is compatible with preclinical data showing opposing effects on neurogenesis of baseline tonic and more intense stimulation of the ECS (33). It is thus likely that the effects of CU reflect these differential effects. Low doses have anxiolytic and antidepressant properties, while high doses are associated with anxiety and depressive symptoms (63).

While these results may be interpreted as indicating that cannabis “causes” depression, there are also data suggesting alternative interpretations, namely that the causal relationship may involve an increased likelihood of CU in individuals with depression. The high rates of lifetime CUD in the population of individuals with MDD (39%) is much higher than in the general population (64). Indeed, depression seems to be a major risk factor for developing symptoms of CUD (65). An epidemiological study in the US described odds for lifetime CUD that were 3.9 times higher for people with mood disorders (including MDD) (65, 66). Similarly, a Canadian study found the 12-month prevalence of CD to be 7-fold higher in those with MDD, while cannabis abuse was 3.5-fold higher (66). In a meta-analysis of the prevalence of comorbid substance use in people suffering from MDD, the point prevalence of CUD was 0.117 (27). In addition, in a community-based study, a one standard deviation increase in depression in adolescence was associated with a 50% increased likelihood of CUD (67, 68).

## Effect of Cannabis on the Age of Onset of Depression

Evidence regarding the effect of cannabis on the age of onset of depression is inconsistent. A population based longitudinal study published in 2017 reported that the onset of depression occurred at a younger age in the non-cannabis using population than in those who used cannabis (64). However, another literature review found that an earlier onset of CU was associated with a shorter time to the emergence of MDD (7). In other studies, this association was no longer significant after controlling for a variety of psychosocial factors (education, alcohol and other illicit drug use and childhood upbringing) (7, 12, 69). The frequency or dose of CU may influence the age of onset of depression. Systematic reviews conducted in 2017 and 2020 found that higher levels of CU were correlated to an earlier onset of depression (18, 57). Several other studies observed this same correlation between heavier CU and early onset of depression (7, 51, 59, 66, 70).

Overall, studies support a bidirectional relationship between depression and CU. In other words, studies support the view that CU is a risk factor for developing depression (52, 54, 57, 58, 71). Moreover, heavier CU is associated with a greater risk of developing depression (29). Inversely, the data also reveals that depression itself is a major risk factor for CU (64, 65). Individuals with depression are also at greater risk of developing CUD (65, 66). A study using a twin-model approach added further evidence of this bidirectional relationship showing an OR for the incidence of MDD in individuals with preceding CUD was 2.54 whereas the OR for the incidence of CUD in those with pre-existing depression was 2.28 (66). Although both no association (16, 18) and reverse directionality (18, 55) have been observed in some studies, this same twin-model study concluded that the model best fitting the data is that of CUD leading to MDD (66). Definitive conclusions regarding the relationship of depression and CU are premature at this stage and data suggest that other factors such as sex, genetic predisposition, personality disorder and psychosocial circumstances may underpin the relationship between CU and depression (6, 51, 59, 72–74).

## Influence of Cannabis on the Course and Clinical Presentation of Depression

In the general population, cannabis use is associated with psychomotor retardation and emotional withdrawal (18, 30), particularly at higher doses. Anxiety, cognitive impairment and addiction to cannabis have also been observed as possible adverse effects of CU (30, 75), although not in all studies (76). CU is associated with poor sleep quality, although this effect may be mediated by concomitant depressive symptoms (77).

Anhedonia is a prominent symptom of depression and engages a broad network of neuronal circuits (78). Cannabis produces a widespread reduction of brain activity, as well as more specific reductions in the ventral striatum (nucleus accumbens) and orbitofrontal cortex in response to reward (6, 79). Liu et al. described a similar alteration in the function of the nucleus accumbens in patients with MDD (80). CU as a contributor to anhedonia has been proposed as a path whereby CU may contribute to depression (5, 78). Several studies have reported apathy and anhedonia in cannabis users (81–83), while others failed to detect this phenomenon (84–86). Decreased cerebral activation in response to reward is reduced in cannabis users, and more so in those with recent heavy CU (87). Although CU may contribute to anhedonia, additional data indicate that anhedonia in adolescence may predispose to CU (88). Since apathy and anhedonia are also seen in depression, one can theorize that the effects of CU may overlap with the symptoms of depression, leading to their exacerbation or potentially confounding the diagnosis of MDD. Although anhedonia can be seen as the result of cannabis-induced inflammation (34), a recent review concludes that the ultimate effect of cannabis is anti-inflammatory (89). Decreased dopamine activity, as seen with chronic CU (44), has also been proposed to be a cause of anhedonia in depression. Since low doses of cannabis enhance dopamine synthesis, anhedonia would not be manifested among those who restrict their CU to modest concentrations (44). Exploration of the interaction of CU and anhedonia in individuals with depression may help to elucidate this interaction.

In individuals with MDD, CU and CUD are associated with having more symptoms than in individuals with MDD who do not use cannabis. These symptoms include anhedonia, changes in weight and sleep, as well as psychomotor changes (1, 64). Another longitudinal study found that CU worsened the symptoms of depression and anxiety, and was associated with poorer mental health and functioning (71).

CU seems to have prognostic implications. Evidence from a population-based longitudinal study in individuals with baseline depressive disorder and varying levels of cannabis usage showed that there was a significant association between the level of CU and the persistence of depressive symptoms at follow-up. However, remission of MDD was not significantly different between those with CU, CUD, or no use (64). A large prospective cohort study showed an association of cannabis use with more depressive symptoms at a 3-year follow-up. Again, no correlation was found regarding the rates of remission, nor was any correlation found with functional impairment (57). CUD in the 6 month period prior to treatment is associated with an increased risk of treatment resistance in depression (3). Overall, the available data points, albeit inconsistently, in the direction of an association of CU and CUD with poorer outcomes in individuals with depression.

Results from different studies are inconsistent with regards to the suicidal risk associated with cannabis in individuals with MDD. In one study, the OR associated with suicidal ideation in people from the general population using cannabis compared to non-users was 1.50 (58). A Canadian populational study found that those who used cannabis at least once a month had a 1.55-fold OR of reporting suicidal ideation in 2012 compared to 2002 (53). In an analysis of the same data, an association between CU and suicidal ideation and attempts was apparent for women but not for men (29). Gobbi et al. noted an increased risk of suicidal attempts in cannabis users compared to non-users with an OR of 3.46 (58). A twin study involving 13,986 individuals found CU to be associated with MDD, suicidal ideation, suicidal plan and attempt (61). Several reviews conclude that CU in adolescence is a harbinger of later, variously defined, suicidal tendencies (6, 51, 61, 72). In contrast to the data pertaining to suicidal ideation, Naji et al. did not find an association between CU in individuals with mood disorders (bipolar disorder and depression) and suicide attempts (90), nor Ostergaard et al. between CUD and suicide attempts or completed suicide (91). Two reviews (18, 57) and a populational study (64) failed to document significant changes in suicidal ideation or behavior in people with MDD after adjusting for confounding factors. Finally, a study by Hesse et al. found that compared to the general population, suicide was actually less frequent in individuals with CUD who received treatments in centers for substance use disorders (HR = 0.69) (92). In all, the preponderance of evidence suggests that cannabis use is not associated with suicidal ideation, suicide attempts or completed suicide in MDD.

## Effects of Cessation of Cannabis Use

Cannabis withdrawal can occur amongst regular or heavy users at cessation. The reasons that motivate CU may vary (4). Using cannabis recreationally positively reinforces use. However, negative reinforcement also drives CU in order to avoid the withdrawal symptoms which emerge following the reduction or cessation of CU (4). Symptoms associated with stopping regular cannabis consumption include depressed mood, anxiety and sleep problems, among others (93). These symptoms may be mistaken for an exacerbation of depression. On the other hand, some studies show that a reduction in CU and cannabis abstinence are associated with improvements in anxiety, depression and functioning in individuals with problematic CU (14, 94). As such, these observations are consistent with the idea that mood symptoms may be secondary (not antecedent) to CU. A randomized controlled trial studying young female adults with depression found that reducing the consumption of cannabis improved mood (3).

At the neurobiological level, CB1 receptor density in the frontolimbic system has been shown to be lower in people consuming cannabis regularly. Those alterations with daily CU are reversed following a month of abstinence (8, 95). This implies that it is necessary to maintain cannabis cessation for at least a month before evaluating its impact on clinical symptoms. Eisen et al. evaluated 56 twin pair members who had either used cannabis (average of 1,085 days) or had not used cannabis (average of 5 days) (96). There were no significant differences in mental health symptoms between the two groups 20 years after their last use (96), suggesting a lack of long-lasting residual effects.

## Influence of Cannabis on Cognition

The impacts of cannabis on cognition in the general population are more fully described in another article in this issue. By comparison, there is a dearth of knowledge regarding the effect of CU on cognition in depression. Cognitive complaints feature among the commonly reported side effects of CU (31). Briefly, in the general population, acute effects of cannabis on cognition include moderate deficits in working memory, verbal learning, and smaller impairments in attention and speed of processing (5). These findings are in line with findings of cannabis-associated altered cerebral function. For example, Lorenzetti et al. found abnormal activity in the frontal-parietal network of adolescent cannabis users (97). Of 13 studies, 10 found differences between cannabis users and controls. The most consistent regions affected were the inferior parietal and the anterior cingulate cortex. Although this review found changes in brain activity in chronic users of cannabis, attributions are complicated by comorbidities, a lack of information regarding the degree of use of cannabis and the varying tasks used during functional imaging. Nevertheless, the implication of the anterior cingulate cortex and the hippocampus highlights commonalities with depression.

Cannabis use is also associated with residual impairment in cognitive performance in healthy individuals (12, 18), in particular memory deficits, and verbal memory (98, 99). Schreiner and Dunn confirmed a small but significant negative effect of CU on cognitive function. However, when the analysis was limited to those studies that required at least 1 month of abstinence, no decrement in cognitive function was detected (95). The amplitude of cannabis-induced cognitive alterations may vary according to dose and age of onset. Acute and chronic CU has an impact on cerebral function and CU, particularly in adolescence, leads to changes in brain structure (41). Likewise, in the large, longitudinal studies performed thus far, deficits in attention, speed of processing and verbal memory have been observed, most particularly in the case of chronic, persistent, cannabis use initiated during adolescence (100). Heavy use of cannabis in adolescents has been shown to produce decrements in attention, learning and processing speed which resolve within 3 months after cessation (101). Preclinical research shows that the administration of THC to adolescent mice generates changes in 5-HT6 (a serotonin receptor) by activating a signaling system, known as the mechanistic Target of Rapamycin (mTOR). This exposure is associated with cognitive deficits in adulthood (102). This same pathway has also been implicated in depression (103) and provides an intriguing physiological mechanism whereby THC consumption in adolescence may contribute to depression vulnerability.

Another factor to consider is that the effects of THC and CBD on cognition may be in opposite directions. However, this is as yet unproven (5). Furthermore, according to a systematic review on the effects of CU on cognition, brain structure and function, chronic CU was associated with changes in hippocampal volume and gray matter density, although the magnitude of the effect was relatively small (104). Similarly, a meta-analysis of task-based fMRI studies on the residual effects of cannabis showed an association between the level of cannabis use and impaired activity of the hippocampus (105). The hippocampus plays a key role in episodic memory (106), a cognitive domain that has been shown to be consistently impaired by acute and chronic cannabis use. Noteworthy, the cognitive impairment associated with cannabis in regular users may not be long lasting. Indeed, a review detected deficits 7 days after heavy use but less consistently beyond that point (104). A recent study showed recovery of cognition 2 weeks after cessation of CU (107). Nevertheless, in those who began CU before age 18, impairment could be detected as long as a year after cessation of consumption (104).

Cognitive deficits are ubiquitous in MDD (6, 98). Of moderate amplitude, these deficits include decrements in executive function, working memory, and attention (108–110). Changes in cognition may be seen as early as the first episode of depression (111) and may persist upon remission. Interestingly, structural brain changes in depression in the hippocampus and density of gray matter in some cortical regions are similar to those seen in individuals who use cannabis regularly. Changes in volume and cortical thickness in several brain regions (hippocampus, anterior and posterior cingulate gyrus, frontal and temporal lobes) may underlie the cognitive deficits of depression (112). Observations of decreased neurogenesis in the hippocampus and its reversal by antidepressants have led to the theory that changes in neuroplasticity are central to the pathogenesis of depression as well as its treatment (113, 114).

Knowledge is sparse regarding the interactions of the cognitive deficits of MDD and those linked to CU. The cognitive deficits linked to CU and MDD may be additive, especially those involving verbal learning (98). However, other data suggests that cannabis users who are not depressed have greater cognitive impairment than individuals with depression who use cannabis (115). Observations from a third study show similar deficits in verbal learning with cannabis use irrespective of the presence of depression (116). These contradictory findings are difficult to reconcile. More research is required on the impact of cannabis on cognition in individuals with MDD.

## Treatment Considerations

Preclinical studies show that antidepressant treatments [desipramine, imipramine, fluoxetine, citalopram, tranylcypromine and electroconvulsive therapy (ECT)], modulate the ECS (6, 63, 117). ECT and imipramine, a tricyclic antidepressant, increase CB1 receptor density in subcortical limbic structures (hippocampus, amygdala, hypothalamus) (30, 63, 117). In addition, sleep deprivation, an intervention that is effective for the treatment of depression, also increases CB1 receptor signaling (33). Long-term treatment with antidepressants and ECT decreases basal stress-induced hypothalamic pituitary adrenal axis (HPA) activation, and increases levels of BDNF as well as neurogenesis (33). This body of evidence suggests that cannabis could have a therapeutic effect on depression. Unfortunately, there is a dearth of evidence addressing this issue.

The quality of evidence concerning the use of medical marijuana in the treatment of psychiatric disorders such as depression is low (118). To our knowledge, no randomized controlled trials have been conducted on the effect of medical marijuana on depression as a primary outcome (57, 119, 120). Preclinical data suggests that CB1 receptor ligands may modulate and potentially enhance the effects of antidepressants (121). An important observation is that CB1 receptor activation can have both depressant and anti-depressant activity (122). This may explain, at least in part, the contradictory results found in the literature of the interactions of cannabis and depression.

Clinical trials using medical marijuana and its by-products for other psychiatric and medical conditions, which included depression as a secondary outcome, have generated intriguing signals. For instance, it was found that the oral administration of nabiximols (an oromucosal spray containing a mixture of THC and CBD) (123) for numerous medical conditions had no significant effect on depression, when studied as a secondary outcome (57, 119, 120). Similar results were observed with dronabinol (an isomer of THC) (119, 124). Moreover, in a randomized, double-blind, placebo-controlled clinical trial for the treatment of neuropathic pain with the nabiximol Sativex, there were no significant modifications in measures of depression and anxiety (43). In fact, a study comparing different doses of nabiximols to placebo found out that the use of a high dose (11–14 sprays/day) exacerbated depression (119), reinforcing the signal that higher doses of cannabinoids may be pro-depressogenic. In contrast, early data from pre-clinical studies of CBD are suggestive of possible antidepressant effects (125–129). We are unaware of any randomized controlled trial investigating nabilone (synthetic orally administered THC compound) or CBD in the treatment of MDD. Finally, while CBD has been proposed to reduce the negative psychoactive effects of THC, a recent study and meta-analysis did not find support for this proposition (130, 131).

There has been little research into the treatment of CUD and comorbid MDD and the available data did not signal any efficacy for pharmacological treatment (132, 133). Several studies of psychosocial interventions have been performed in patients with severe mental illness and CUD. However, apart from a few preliminary trials (70, 134), these studies have not focused specifically on MDD (135).

It is premature to recommend cannabis or its derivatives as a treatment for depression. A recently published review of promising preclinical evidence detailing CBD's potential as a therapeutic agent concludes with a call for further research into CBD's clinical efficacy (129). The American Psychiatric Association has concluded that “*There is no current scientific evidence that marijuana is in any way beneficial for the treatment of any psychiatric disorder. In contrast, current evidence supports, at minimum, a strong association of cannabis use with the onset of psychiatric disorders”* (22).

As discussed in this article, there is evidence linking THC with worsening of the symptoms of depression, and also a suggestion that CBD may be associated with favorable effects when used to treat depression. This information can be used to steer patients with depression away from the use of high THC content cannabinoid products, particularly during adolescence.

## Future Directions

In the recent context of legalization, and the availability of cannabis characterized by higher concentrations of THC and lower concentrations of CBD, there exists an urgent need for well-designed studies on the benefits and harms of medicinal and recreational cannabis and related compounds in major depression.

In epidemiological and clinical studies, the exposure to cannabis should be more precisely defined both in terms of frequency and quantity of use in prospective studies that do not have to rely on recollection for this information. Comorbid substance use, and comorbid medical and psychiatric conditions should be documented, as they may confound findings that could be erroneously attributed to CU.

In order to clarify the role of cannabinoids as therapeutic agents for the treatment of depression, studies with this aim as a primary outcome are essential. Well-designed, appropriately powered, studies of the pharmacological treatment of MDD and comorbid CUD are essential. Trials of the efficacy of cannabis or its derivatives in MDD should have appropriate strategies for concealment and include a placebo control. The populations studied should be clearly defined and the diagnosis of MDD established through appropriate diagnostic evaluations. It is essential to examine dose-response relationships and the influence of cannabis composition (e.g., THC/CBD ratio) on treatment. Low doses of cannabis or its derivatives should be tested, as there is a clear signal that there is a different pharmacological effect of high and low dose. Future research should consider that this complex molecule also has the potential for drug-drug interactions (136–139). The dimensions of apathy, anhedonia, cognition and anxiety will be important secondary outcomes to consider.

For those who suffer from MDD and comorbid CUD, there is an urgent need to investigate, in well-designed trials, the potential efficacy of motivational interviewing and cognitive behavioral therapy. Such interventions have been shown to be efficacious for the treatment of CUD in individuals with no major psychiatric disorder. It remains to be determined if these interventions are also efficacious in individuals with MDD and CUD.

## Limitations

This review was not systematic and did not restrict the definition of depression to a clinical diagnosis of MDD. Some articles used cut-off scores on scales to define depression. Further, the literature presents inconsistent results, which may be a consequence of the lack of precision regarding the concentrations of THC, CBD, and the strains consumed. Finally, some of the studies were small and thus, their results may not be generalizable.

## Conclusions

CU, in particular of cannabis products higher in THC content, is likely to be associated with increased adverse psychiatric effects, including depression. Indeed, meta-analyses on the subject seem to show that cannabis use may be a risk factor for the development of depression. However, a bidirectional relationship has also been described with depression being a risk factor for cannabis consumption as well as the reverse. Gender and youth may confer increased vulnerability to the adverse effects of cannabis.

There is evidence that the endocannabinoid system is involved in the pathophysiology of depression. In the future, larger studies in the field will be needed to demonstrate this involvement, especially positron emission tomography studies examining different components of the endocannabinoid system. Components of this system are clearly potential targets for new therapeutic interventions for depression.

Preliminary evidence from clinical trials shows that low doses of cannabis and its products have different and potentially beneficial effects, in contrast to higher doses which are associated with adverse effects. While some preliminary data indicates less deleterious and possibly positive effects of CBD in depression, it is premature to recommend CBD as a treatment for depression (30). RCTs on this topic are warranted. Finally, in considering the use of cannabis and its derivatives, it is important to balance the possible alleviation of anxiety and depression against side effects such as apathy and cognitive deficits.

## Author Contributions

CL and ST were central to gathering data and writing the manuscript. AK and SP reviewed the manuscript. All authors contributed to the article and approved the submitted version.

## Conflict of Interest

AK has consulted with Spectrum Therapeutics and Tilray in an advisory, mentorship and educational role. The remaining authors declare that the research was conducted in the absence of any commercial or financial relationships that could be construed as a potential conflict of interest.
